# Whole-genome assembly of *Culex tarsalis*

**DOI:** 10.1093/g3journal/jkaa063

**Published:** 2021-01-11

**Authors:** Bradley J Main, Matteo Marcantonio, J Spencer Johnston, Jason L Rasgon, C Titus Brown, Christopher M Barker

**Affiliations:** 1 Department of Pathology, Microbiology and Immunology, University of California, Davis, CA 95616, USA; 2 Department of Entomology, Texas A&M University, College Station, TX 77843, USA; 3 Department of Entomology, The Center for Infectious Disease Dynamics, and the Huck Institutes of the Life Sciences, University Park, PA 16802, USA; 4 Department of Population Health and Reproduction, University of California, Davis, CA 95616, USA

**Keywords:** Genome, mosquito, Vector, Genetics, PacBio

## Abstract

The mosquito, *Culex tarsalis*, is a key vector in the western United States due to its role in transmission of zoonotic arboviruses that affect human health. Extensive research has been conducted on *Cx. tarsalis* ecology, feeding behavior, vector competence, autogeny, diapause, genetics, and insecticide resistance. Population genetic analyses in the western U.S. have identified at least three genetic clusters that are geographically distinct. However, in-depth genetic studies have been hindered by the lack of a reference genome. In this study, we present the first whole-genome assembly of this mosquito species (CtarK1) based on PacBio HiFi reads from high-molecular-weight DNA extracted from a single male. The CtarK1 assembly is 790 Mb with an N50 of 58 kb, which is 27% larger than *Culex quinquefasciatus* (578 Mb). This difference appears to be mostly composed of transposable elements. To annotate CtarK1, we used a previously assembled *Cx. tarsalis* transcriptome and approximately 17,456 protein genes from *Cx. quinquefasciatus* (*N* = 17,456). Genome completeness was assessed using the Benchmarking Universal Single-Copy Orthologs (BUSCO) tool, which identified 84.8% of the 2799 Dipteran BUSCO genes. Using a Bayesian phylogeny based on mitochondrial genomes, we place *Cx. tarsalis* in the context of other mosquito species and estimate the divergence between *Cx. tarsalis* and *Cx. quinquefasciatus* to be between 15.8 and 22.2 million years ago (MYA). Important next steps from this work include characterizing the genetic basis of diapause and sex determination in *Culex* mosquitoes.

## Introduction

*Culex tarsalis* is one of the most important vector species in the western United States of America due to its capacity to transmit arboviruses that cause disease in humans and horses (Turell *et al.* 2005). This mosquito is the principal vector for West Nile virus in agricultural areas (Goddard *et al.* 2002; Reisen 2013) that have the highest incidence of West Nile virus disease. Extensive research has been done on *Cx. tarsalis* ecology (Reisen 2012), feeding behavior (Thiemann *et al.* 2012; Reisen *et al.* 2013), vector competence (Kramer *et al.* 1981; Reisen *et al.* 2006), autogeny (Spadoni *et al.* 1974; Reisen 1995), diapause (Reisen 1986; Buth *et al.* 1990; Reisen *et al.* 1995), and insecticide resistance (Ziegler et al. 1987). A genetic linkage map has also been developed (Venkatesan *et al.* 2009) and three genetically distinct populations have been described in the western U.S., namely: the Pacific, Sonoran, and Midwest genetic clusters (Venkatesan and Rasgon 2010). However, the lack of a published reference genome for *Cx. tarsalis* has likely hindered basic and applied science involving this species. For example, genomic studies in *Anopheles* mosquitoes have yielded important insights into sympatric speciation (Turner *et al.* 2005) and characterizing the genetic basis of complex insecticide resistance has led to development of improved vector control technology, like PermaNet 3.0 (Tungu *et al.* 2010). Innovative gene-drive-based mosquito control methods, including population replacement (Gantz *et al.* 2015; Carballar-Lejarazú *et al.* 2020) and population suppression (Adelman and Tu 2016) approaches are also now being developed for *Aedes* and *Anopheles* mosquitoes, both of which have high quality reference genomes.

Here we describe the first genome assembly for *Cx. tarsalis*, including annotations of the pyrethroid resistance allele referred to as knockdown resistance (*kdr*) in the voltage-gated sodium channel (VGSC) gene*.* The assembly is based on PacBio HiFi reads from high-molecular-weight (HMW) DNA from a single adult male and 10X Genomics linked-reads (10X) were used for assembly of the mitochondrial genome. We used DNA from a male as input so that the dominant sex locus could be identified as well as the female sequence at that region. This *Cx. tarsalis* reference genome will facilitate the development of insecticide resistance genotyping assays, GWAS, characterization of sex determination, and comparative genomics studies among *Culex* mosquitoes.

## Materials and methods

### Mosquitoes

All *Cx. tarsalis* used for the genome assembly were sampled from the Kern National Wildlife Refuge colony (KNWR; 35.7458°N, 118.6179°W), which was established in 2002. For PacBio sequencing, HMW DNA was extracted at the UC Berkeley DNA Sequencing Facility from a single male adult. To correct PacBio sequencing errors, we prepared and sequenced two Nextera libraries (Illumina) with paired-end 75 bp reads from another single adult male and single adult female. For 10X library preparation, HMW DNA was extracted by the UC Davis DNA Technologies Core from two late-eclosing, relatively large pupae (likely female). Two pupae were used for 10X because pure HMW DNA yields were too low from a single individual.

### PacBio genome assembly

PacBio sequencing was performed on a Sequel II SMRT cell at the UC Berkeley sequencing core. Circular consensus sequences (CCS) were then generated and filtered with high stringency to get HiFi CCS reads. We generated an initial genome assembly using Canu v1.9 with the following settings: genomeSize = 0.875g, useGrid = false. The genome coverage was low (mode = 8x), but because these were HiFi reads, we reduced the standard minimum coverage thresholds with the following options: stopOnLowCoverage = 1, contigFilter = “2 0 1.0 0.5 0”. The PacBio mitochondrial contig appeared to be two full mitochondrial genomes stuck end-to-end. As a result, we removed this contig and replaced it with the full mitochondrial genome generated from the 10X assembly. Then we performed genome polishing using racon (v1.4.3) and 75 bp Nextera reads (Illumina) from a single adult male from the KNWR colony. Additional SNPs in the reference sequence compared to multiple sequenced KNWR individuals were removed by extracting the consensus sequence from a single male KNWR library mapped to the reference using bcftools. Annotations were performed using MAKER (v.2.31.10) with the *Cx. tarsalis* transcriptome (Ribeiro *et al.* 2018) and *Culex quinquefasciatus* protein sequences (CpipJ2.4). Repeat masking was performed in parallel using the MAKER annotation pipeline. As input, we used the standard RepBase database (RepBaseRepeatMaskerEdition-20170127) and a *Cx. tarsalis*-specific repeat library that was generated using RepeatModeler (v1.0.11). The mitochondrial genome and the VGSC gene were re-annotated using Geneious software and the *Cx. quinquefasciatus* mitochondrial genome (NC_014574) as a reference. To calculate genome statistics, including the N50, we used Quast (v5.0.2).

### 10X genomics linked-read genome assembly

The HMW DNA extraction, 10X Genomics Chromium sequencing library, and Illumina sequencing was performed at the UC Davis DNA Technologies Core. Raw linked reads were assembled using the Supernova 2.0.1 software (10X Genomics) on an Amazon Web Services instance with 480 Gb on RAM and 64 logical cores. To generate a fasta formatted reference sequence, we used supernova mkoutput with –style = pseudohap. This option arbitrarily selects haplotypes across the genome resulting in one pseudo-haploid assembly composed of a mosaic of paternal and maternal haplotype stretches.

### Phylogenetic analyses

The mitochondrial analysis included 15,203 bp of mitochondrial sequence from each species: *Cx. tarsalis* (CtarK1, this study), *Cx. quinquefasciatus* (NC_014574), *Anopheles gambiae* (NC_002084), *Anopheles coluzzii* (NC_028215), *Aedes aegypti* (NC_035159.1), *Aedes albopictus* (NC_006817), and *Drosophila melanogaster* (NC_024511). A multiple sequence alignment was performed using MUSCLE (v3.8.425; (Edgar 2004)) and then the phylogenetic analysis was performed using the Bayesian Evolutionary Analysis by Sampling Trees software (BEAST) v 2.6.2 (Drummond and Rambaut 2007). To determine the best combination of substitution and clock models, model selection was performed through generalized stepping-stone sampling (GSS) using BEAST Path Finder tool (1 M iterations and 100,000 pre-burnin). The model with the lowest marginal log-likelihood had generalized time-reversible substitutions (GTR, with 4 category count for the Gamma site model), relaxed log-normal clock, and Yule tree model. In addition, to improve the estimated divergence time for the *Culex* clade, we used a Yule calibrated tree model using the following previously published tree calibration constraints: 71 (44.3, 107.5) million years ago (MYA) for *Ae. aegypti* and *Ae. albopictus*, 179 (148.0, 216.7) MYA between *Culex* and *Aedes*, 217 (180.8, 256.9) MYA between *Culicinae* and *Anopheles*, and 260 (238.5, 295.5) MYA between *Drosophila* and *Culicidae* (Chen *et al.* 2015)*.* All clades with priors were considered monophyletic. The final phylogenetic tree was generated running 100 M iterations with 10 M burn-in saving samples every 1000 steps in order to obtain a representative sample [*i.e.*, estimated sample size (ESS) higher than 200 for all important model parameters] for the posterior distribution of model parameters. The maximum clade credibility tree with average node heights was generated in TreeAnnotator v 2.6.0, by removing the initial 10% fraction of the chain.

### Genome size estimate

Genome size was estimated as described in Johnston *et al.* (2019), and is based on the fluorescence scored using a Partex CX flow cytometer that was equipped with green laser excitation. Briefly, the head of a single *Cx. tarsalis* mosquito was combined with the head of a *Drosophila virilis* standard (1 C = 328 Mbp) in 1 ml of cold Galbraith buffer and ground with 15 strokes of the “A” pestle in a 2 ml Kontes Dounce. The nuclei released were filtered through a 45 U nylon mesh, stained for 1 hour in the cold and dark using 25 μg/ml propidium iodide. The total mean fluorescence of the 2 C (diploid) peaks from the sample and standard was measured as a mean channel number using the software supplied with the Partex CX. The 1 C amount of DNA in the mosquito was estimated as (mean channel number of the 2 C peak of *Cx. tarsalis*/mean channel number of the 2 C *D. virilis* peak) X 328 Mbp. More than 2000 nuclei were scored under each 2 C peak and the CV of the peaks were all <2.5.

### Data availability

The reference genome, annotation file, and scaffold sequences flanking the sex locus were deposited at the open science framework https://osf.io/mdwqx/.

Supplementary material is available at *G3* online.

## Results and discussion

### Genome assembly

The PacBio long-read library generated from a single adult male *Cx. tarsalis* was sequenced on the Sequel II platform and yielded 5.7 M raw reads and 988,512 ccs HiFi reads. The HiFi reads were used to prepare a draft assembly using Canu with reduced filtering thresholds because at this stage in the *Cx. tarsalis* genome assembly, we were willing to accept some sequence errors in order to increase genome representation from PacBio reads. This resulted in 19,994 contigs and a total genome size of 789,669,425 bp (Quast v5.0.2). The N50 was 57,901 bp, the GC content was 36%, and the largest contig was 753,184 bp (Table 1). To assess the genome completeness of the CtarK1 PacBio assembly, we searched for the presence of a set of 2799 Dipteran Benchmarking Universal Single-Copy Orthologs ( BUSCO; (Zdobnov *et al.* 2017; Waterhouse *et al.* 2018). We detected 79% (2219/2799) as complete single-copy genes, 8% (227/2799) as complete and duplicated, 5% (153/2799) were fragmented, and 15.2% (427/2799) were missing.

**Table 1:** Assembly statistics

Assembly	Genes	Contig #	Median contig (N50)	Total length
*Cx. quinquefasciatus* (CpipJ2.4)	19,793	3,172	486,756 bp	∼579 Mb
*Cx. tarsalis* (CtarK1)	17,456	19,994	57,901 bp	∼790 Mb

Using flow cytometry, we estimated the complete haploid genome size of *Cx. tarsalis* to be 890 Mb. This is over 50% larger than *Cx. quinquefasciatus* genome (579 Mb, see Table 1). This genome size estimate and the BUSCO scores from our 790 Mb PacBio assembly indicate that this initial assembly is approximately 85–90% complete. Additional long-read sequencing from single individual mosquito input and genetic scaffolding (*e.g.*, with Hi-C) is needed to make CtarK1 comparable in quality to other model species.

For the mitochondrial genome, we used a 10X chromium library that was sequenced on Illumina’s Novaseq platform, yielding approximately 507 million clusters (paired-end reads) passing filter. Based on several trial assemblies, we downsampled the total read input to 350 million paired-end reads to yield approximately 56x coverage. A contig containing the complete mitochondrial genome was then identified, and redundant sequence (due to circular genome) was trimmed at each end of the contig. While we included the 10X mitochondrial genome in our CtarK1 assembly and we used 10X contigs to make scaffolds at the sex locus (described later), we avoided making a consensus genome between PacBio and 10x because of concern over high levels of redundancy in 10X assembly. For example, the final 10x assembly size (1.5 Gbp) was nearly twice the expected size (890 Mb). This was likely a result of inputting multiple pupae, which resulted in multiple haplotypes in the assembly.

### Genome annotation

A total of 14,726 complete genes were identified by the maker annotation pipeline (see methods). An additional 2730 gene orthologs have support from protein2genome annotations with an arbitrary overlap threshold of 80% or less. Thus, we identified approximately 17,456 genes in CtarK1. While this is substantially fewer than the number for *Cx. quinquefasciatus*, direct comparisons should not be made until BUSCO scores are similar between the assemblies.

Using Repeat Masker and a custom repeat library, a total of 60.8% of CtarK1 was annotated as a repeat feature. This is double the estimate from *Cx. quinquefasciatus (*Arensburger *et al.* 2010*)*, indicating that transposable elements make up a significant portion of the *Cx. tarsalis*-specific genome expansion (800 Mb vs 579 Mb).

### Voltage-gated sodium channel annotations

**Figure 1:**
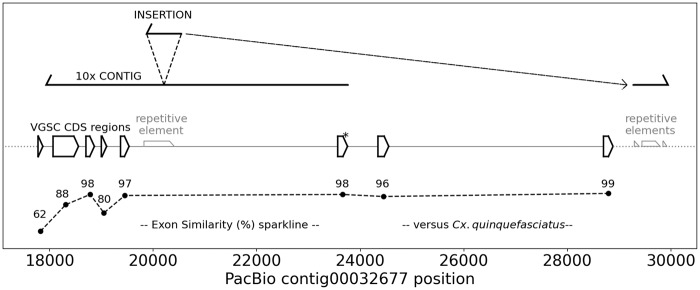
PacBio contig spans the entire voltage-gated sodium channel gene. Annotations of a portion of the VGSC insecticide resistance gene (ortholog of CPIJ007595) on tig00032677 of CtarK1 including coding sequences (open pentagons), the location of *kdr* in exon 6 (*), and the percent similarity of each coding region compared to *Cx. quinquefasciatus* (–). In contrast to PacBio, the corresponding 10X Genomics contig ended after exon 6 and included a large insertion originating from downstream of the VGSC gene. The repeat elements from left to right are: REP-1_CQ, DNA8-14_CQ, rnd-5_family-476, and DNA8-4_CQ.

The emergence of resistance to pyrethroid insecticides in *Cx. tarsalis* is an important issue that needs to be considered and managed for effective vector control efforts. The *kdr* mutation occurs in exon 6 of the sodium channel protein in *Cx. quinquefasciatus* (CPIJ007595; Kothera *et al.* 2019) and we used this gene to annotate *kdr* and flanking exons in *Cx. tarsalis* (see asterisk on Figure 1). We identified a total of eight exons, spanning 11,104 bp, of the VGSC gene in *Cx. tarsalis*. The complete CPIJ007595 gene was not identified by the maker pipeline, but support for multiple exons from *Cx. quinquefasciatus* protein alignments were included in the output. To improve the annotations of the VGSC gene coding regions in tig00032677, we used the *Cx. quinquefasciatus* supercont3.182 (contains CPIJ007595) and associated annotations as input for the Geneious annotation software. Using this approach, eight exons were identified. Most of the exons had high percent similarity between *Cx. tarsalis* and *Cx. quinquefasciatus* (up to 99%), but exon 1 was the most divergent with 62.4% similarity (Figure 1).

It should be noted that CPIJ007595 is only 544 amino acids and complete VGSC genes in *Aedes aegypti* (AAEL013277), *Anopheles gambiae (*AGAP004707), and *Drosophila* melanogaster (CG9907) all code for over 2000 amino acids. Thus, while there are sufficient annotations to facilitate the design of *kdr* genotyping assays, the VGSC gene annotations are incomplete in Ctark1. A major motivation for pursuing PacBio sequencing was that the 10X contigs did not include sufficient flanking sequence to assist with PCR-based assay designs targeting *kdr*. In contrast, the PacBio assembly generated a 31,996 bp contig (tig00032677) that spanned *kdr* by over 8 kb. Further analysis of the 10X contig versus the PacBio contig revealed a 671 bp insertion in the 10X contig that maps in the reverse-complement direction downstream of the VGSC gene. There are several distinct PacBio HiFi reads that span this segment, indicating that this is an error in the 10X contig. Repeat elements at the insertion site and at the downstream mapping position may have contributed to this 10X assembly artifact (Figure 1).

### Sex locus

**Figure 2:**
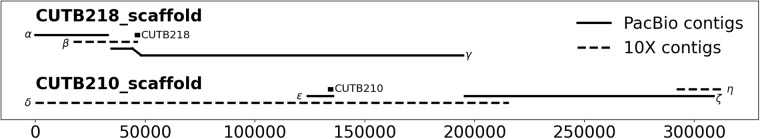
Scaffolding of 10X and PacBio contigs flanking the sex locus. In an attempt to sequence across the sex locus, 10X Genomics (dashed lines) and PacBio (solid lines) contigs were combined at the previously described markers (black squares) that flank the sex locus (Venkatesan *et al.* 2009) on the centromeric (CUTB218) and telomeric (CUTB210) sides. Contigs in the CUTB218_scaffold are as follows: α = PacBio_tig00011961, β = 10x_233502, γ = PacBio_ tig00000802. Contigs in the CUTB210_scaffold are: δ = 10X_430, ε = PacBio_ tig00029712, ζ = PacBio_ tig00005857, and η = PacBio_ tig00005857.

In an attempt to annotate the sex locus in *Cx. tarsalis*, we utilized both the 10X and PacBio contigs to generate scaffold sequences near the sex locus on Chromosome 3. Molecular markers flanking the sex locus have been previously described, including the microsatelite markers CUTB218 and CUTB210 that flank the sex locus by 1.5 cM and 1.4 cM, respectively (Venkatesan *et al.* 2009). The CUTB218 sequence is 481 bp and resides on the centromeric side of the sex locus. This sequence failed to map to CtarK1. However, CUTB218 maps to the extreme end of the contig 10X_233502 (Figure2β) with approximately 20 more CT tandem repeats than the 10X sequence. Using 10X_233502 as a reference, we identified a 32.8 kb PacBio contig PacBio_tig00011961 (Figure 2α), which maps to the first 16,575 bp. Several large insertions were identified in the 10X versus PacBio contigs, including one 5 kb stretch of N’s in the 10X contig. Another large PacBio contig (PacBio_tig00000802, Figure 2γ) mapped to the opposite end of 10X_233502. Approximately 9.5 kb of PacBio_tig00000802 overlapped at the end of 10X_233502; however, the last 2.5 kb (which includes CUTB218) did not align (Figure 2). Due to the lack of alignment at the extreme end of 10X_233502, which includes the sequence of interest (CUTB218), we excluded PacBio_tig00000802 from the CUTB218 scaffold. Thus, the final CUTB218 scaffold was 45,566 bp.

CUTB210 (DQ682690) is a 553-bp segment on the telomeric side of the sex locus and maps to a 17 kb contig of CtarK1 at position PacBio_tig00029712:10,209–10,765 (Figure 2ε). CUTB210 has 9 additional “CT” tandem repeats at position 10,529 and 1 SNP at position 10,626 versus our CtarK1 reference. To scaffold contigs across the sex locus, we used a 215.5 kb 10X contig (10X_430, Figure 2δ), which aligns with PacBio_tig00029712 (Figure 2ε) at position 430:123,826–135,535. Then, we identified PacBio_tig00005857 (113 kb), which maps to the last 20 kb of 10X_430 (Figure 2ζ). Contig 10X_305071 maps to the last 16.8 kb of tig00005857, extending the scaffold approximately another 5 kb (Figure 2η). The final CUTB210_scaffold is 321,101 bp. Attempts to align additional contigs using the *Cx. quinquefasciatus* genome were not successful. To facilitate future efforts to characterize the sex locus, we have included these sequences in Supplementary File S1.

### Phylogenetic analysis

**Figure 3:**
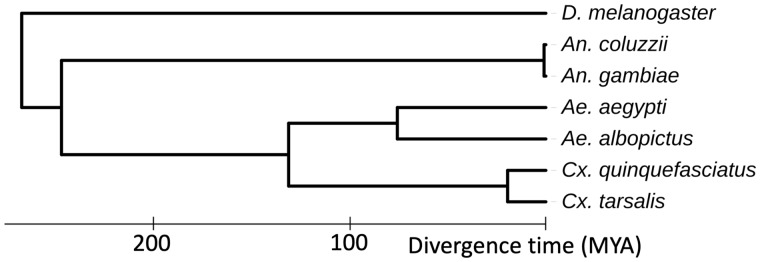
Mitochondrial phylogeny. This Bayesian phylogeny places *Cx. tarsalis* in the context of other sequenced mosquito species and *D. melanogaster* as an outgroup. This estimate was based on multiple mitochondrial sequence alignments (15,203 bp) and calibration constraints for *Aedes* species, between *Culex* and *Aedes*, between *Culicinae* and *Anopheles*, and between *Drosophila* and *Culicidae* (Chen *et al.* 2015). The divergence time estimate between *Cx. tarsalis* and *Cx. quinquefasciatus* is 15.8–22.2 MYA.

Based on multiple-species alignments of complete mitochondrial genomes (15,203 bp), we estimated the placement of *Cx. tarsalis* on a phylogenetic tree with other sequenced mosquito species and *Drosophila melanogaster* as an outgroup. The estimated divergence time between *Cx. tarsalis* and *Cx. quinquefasciatus* was 15.8–22.2 MYA (95% Credible Interval; Figure 3). We also estimated the divergence time between *An. gambiae* and *An. coluzzii* (95% Credible Interval = 0.07–3.45 MYA), which is slightly older than the previous estimate of 0.061 MYA (Thawornwattana *et al.* 2018). The slight difference in the *Anopheles* species divergence is likely due to using mitochondrial instead of whole-genome alignments.

## Conclusions

Here, we present the first whole-genome assembly for *Cx. tarsalis* (CtarK1) based on PacBio HiFi reads from a single adult male. The CtarK1 reference genome is 790 Mb; 27% larger than *Cx. quinquefasciatus* (578 Mb). This difference appears to be mostly composed of transposable elements. To place *Cx. tarsalis* in the context of other mosquito species we assembled a Bayesian phylogeny based on Mitochondrial genome alignments and estimate divergence between *Cx. tarsalis* and *Cx. quinquefasciatus* at between 15.8 and 22.2 million years ago (MYA). Important next steps from this work include assembly of chromosome-level scaffolds using Hi-C, elucidating the genetic basis of adult diapause and sex determination in *Culex* mosquitoes. Understanding these fundamental genetic mechanisms is exciting for evo-devo in insects and may also lead to innovative vector control strategies, including gene-drive-based sex ratio distorters.

## Supplementary Material

jkaa063_Supplementary_Data
